# Surgical Treatment of a Giant Liposarcoma in a Japanese Man

**DOI:** 10.1155/2010/943073

**Published:** 2010-12-16

**Authors:** Yasuhiro Hashimoto, Shingo Hatakeyama, Tokushi Tachiwada, Takahiro Yoneyama, Takuya Koie, Noritaka Kamimura, Takeshi Yanagisawa, Kenichi Hakamada, Chikara Ohyama

**Affiliations:** ^1^Department of Urology, Hirosaki University Graduate School of Medicine, 5 Zaifu-cho, Hirosaki 036-8562, Japan; ^2^Department of Urology, Aomori Rosai Hospital, Hachinohe 031-8551, Japan; ^3^Department of Gastroenterological Surgery, Hirosaki University Graduate School of Medicine, 5 Zaifu-cho, Hirosaki 036-8562, Japan

## Abstract

We report a case of a rapidly progressing giant retroperitoneal liposarcoma weighing 22 kg in a 41-year-old Japanese man, successfully treated with surgical excision. To our knowledge, this is the largest liposarcoma in the Japanese population reported in the literature.

## 1. Introduction

Liposarcoma is a common soft tissue sarcoma which occurs in adults [1, 2]. Its common sites of occurrence are the extremities, retroperitoneum, and inguinal region [3]. Although liposarcomas can often grow into large tumors, resected tumors weighing over 20 kg are extremely rare and considered to be “giant liposarcomas.” Here, we report our experience of a giant retroperitoneal liposarcoma, presumably the largest reported from Japan; it weighed 22 kg and caused abdominal swelling, marked leg edema, and cough due to the pressure it exerted on the patient's diaphragm. 

## 2. Case Presentation

**Figure 1:**
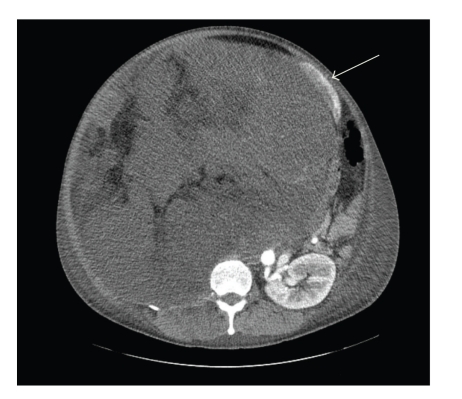
Computerized tomography detected a 43 × 37 × 31-cm tumor in the right retroperitoneum, and the right kidney was deflected greatly to the left ventral side (white arrow).

**Figure 2:**
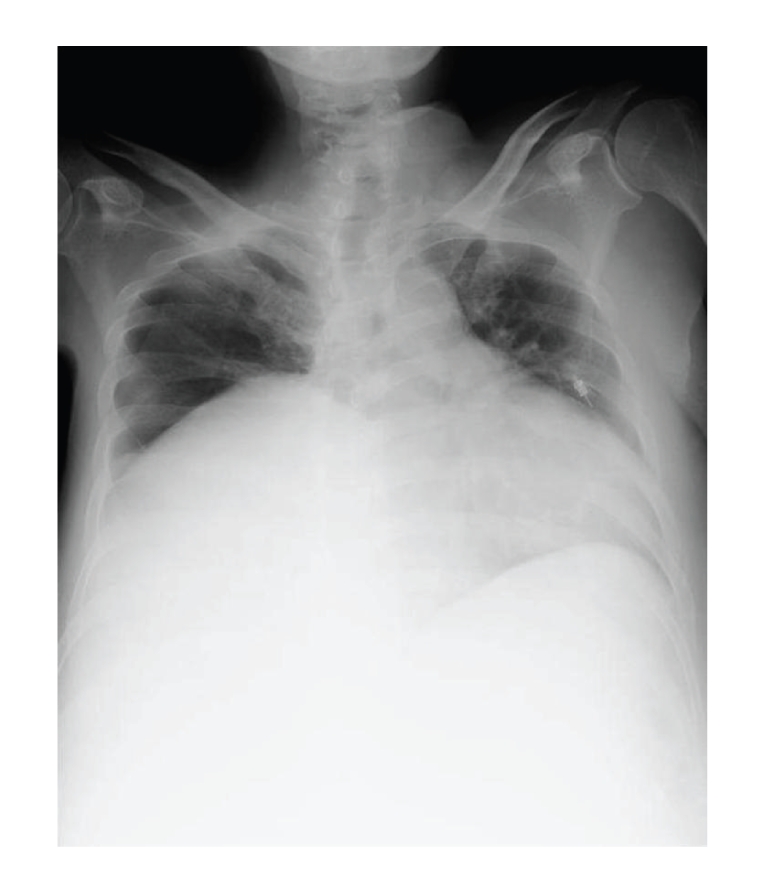
Chest X-ray showed that elevation of the diaphragm due to rapid growth of retroperitoneal tumor and cardiac enlargement.

A 41-year-old Japanese man became aware of abdominal swelling in December 2008 but did not seek a medical opinion until he developed marked leg edema, a 30 kg weight gain, and cough. He was hospitalized the same day after a retroperitoneal tumor was detected by computed tomography (CT). After hospitalization, the patient was diagnosed with a liposarcoma by CT-guided needle biopsy. The patient was emergently transferred to our hospital due to worsening respiratory status. On admission his body surface area was 2.18 m^2^. A CT scan detected a 43 × 37 × 31 cm tumor in the right retroperitoneum, with the right kidney significantly displaced to the left and anterior side (Figure 1). Whole-body CT scan detected no metastasis. His condition was diagnosed as respiratory failure due to elevation of the diaphragm as a consequence of the rapid growth of the tumor (Figure 2).

We concluded that surgical resection was the only option for saving the patient's life, so we performed a tumorectomy 3 weeks later through a midline incision. The operation took 3 hours and 20 minutes, and the estimated blood loss was 3990 mL requiring 800 mL blood transfusion. During surgery, the tumor and right kidney were completely resected.

**Figure 3:**
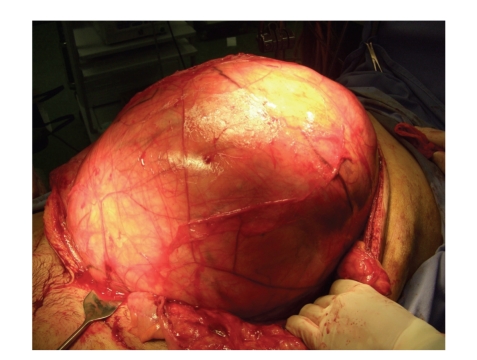
Intra-operative findings. The retroperitoneal tumor weighing 22 kg was extirpated.

**Figure 4:**
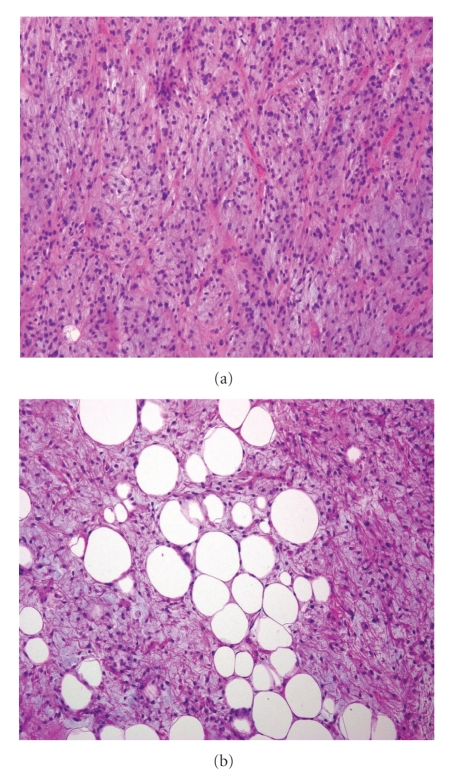
Histopathological findings of the tumor diagnosed dedifferentiated liposarcoma (HE stain, original magnification x200). (a) Most of the tumor cells consisted of spindle cells with high-grade atypia. (b) Some of which were identified to be lipoblasts in a myxoid background.

Pathological examination showed that the resected mass weighed 22 kg and was 45 × 40 × 30 cm in size (Figure 3). Grossly, the tumor was well encapsulated and transverse sectioning revealed a solid multinodular tumor mass. Histopathologic analysis demonstrated many spindle cells with high-grade atypia; some of which were lipoblasts on a myxoid background; there were no split signals on FISH analysis using CHOP DNA probes, thus leading to the diagnosis of dedifferentiated liposarcoma (Figure 4). Microscopically, no tumor cells were identified in the right kidney and this was an R0 resection. He declined adjuvant therapy. The patient was discharged from our hospital after 18 days. His body surface area at discharge was 1.66 m^2^. A CT scan performed 12 months after surgery detected no recurrence.

## 3. Discussion

Liposarcoma is the most common soft tissue sarcoma and accounts for 10–20% of all cases. It commonly occurs between 40–60 years of age and has a 1:1 ratio between genders [1, 2]. Liposarcoma occurs most commonly in the extremities (52%), retroperitoneum (19%), and inguinal region (12%) [3]. Although liposarcomas sometimes weigh as much as 10 kg or more, those exceeding 20 kg are extremely rare. Some published reports have noted tumors weighing 103.6 lb (46.6 kg) [4], 42 kg [5], and 18 kg [6]. According to previous reports, the heaviest tumor to date in the Japanese population was 18 kg [7]. Thus, we believe that we have resected the largest retroperitoneal liposarcoma in Japan.

According to the WHO classification, liposarcomas can be classified into five histologic types: well differentiated, myxoid, round cell, pleomorphic, and dedifferentiated [8]. The myxoid and round-cell types are considered subtypes because of their common chromosomal translocation [9–12].

Dalal argued, on the basis of a study conducted at a single facility, that the most common forms of liposarcoma are well differentiated (46%), dedifferentiated (18%), myxoid (18%), round cell (10%), and pleomorphic (8%). 

A chimera gene, TLS-CHOP, which is only found in myxoid-type sarcomas, was discovered in a myxoid-type liposarcoma. This gene is presumed to be responsible for tumor growth [9–12]. It can often be difficult to distinguish between myxoid-type and dedifferentiated liposarcomas. Nevertheless, we observed no split signals on FISH analysis using CHOP DNA probes.

Surgical resection is still the principal treatment, and combined resection of involved organs is necessary for an R0 resection in many cases. A previous report stated that combined resection of adjacent organs is required in 75% of retroperitoneal tumors treated by tumorectomy [13].

Multimodality therapies such as chemotherapy with ifosfamide, anthracycline, and doxorubicin, as well as the combination of intraoperative and intensity-modulated radiotherapy, are also effective [14]. Recently, Ecteinascidin (ET743), an alkaloid derived from the Caribbean sea squirt (*Ecteinascidia turbinate),* has shown definite activity in soft tissue sarcoma in clinical trials [14]. However, high-grade liposarcoma may benefit from radiotherapy or chemotherapy with a reduction in local recurrence, but with no benefit in overall survival [14]. Histologic grading is important in liposarcoma, with disease-specific survival rates after 5 years of 93% for well-differentiated, 74–92% for myxoid/round-cell, 59% for pleomorphic, and 44% for dedifferentiated forms [15]. 83% of patients with dedifferentiated liposarcomas develop local recurrence, and 30% develop distant metastasis [16]. Here, we report the largest retroperitoneal liposarcoma in Japan. Judging from evidence in previously published reports, we expect a poor prognosis for the patient and will be closely following him for recurrence.
